# Supplementary material for: Fast food over safe food? A qualitative evaluation of a food safety training intervention for street vendors applying the COM-B model in Ouagadougou, Burkina Faso
Source: PLoS One. 2024 Nov 21;19(11):e0313635. doi: 10.1371/journal.pone.0313635 (PMC11581311; doi:10.1371/journal.pone.0313635)
Supplement: S1 Table — (DOCX) [file pone.0313635.s001.docx]

**S1 Table. Training overview with key messages**

| Modules | Key messages |
| --- | --- |
| Module 1. Importance of good hygiene practices | 1.1 Responsibility of vendor to maintain public health through the nature of its work  1.2 Contaminated chicken and/or vegetables cause illness in consumers (diarrhoea/nausea/vomiting) |
| Module 2. Knowledge of microorganisms | 2.1 Micro-organisms from external surfaces contaminate grilled chickens and seasonings.  2.2 Sources of contamination in grilled chickens; person, environment, work equipment, or raw chicken |
| Module 3. Live hicken management | 3.1 Separate sick chickens from healthy chickens  3.2 Arrange suitable cages; well-positioned in relation to the wind and reasonable distance from point of sale |
| Module 4. Slaughtering practices | 4.1 Provide a slaughter space that is easy to clean and disinfect  4.2 Regularly change scalding water  4.3 De-feather/pluck and eviscerate on an easy-to-clean and raised surface  4.4 Separate defeathered chickens from eviscerated chickens  4.5 Separate offal (gizzard and liver) from viscera and feathers  4.6 Dispose viscera and feathers every day  4.7 Separate the scalding fireplace from the grill |
| Module 5. Carcass management | 5.1 Wash carcasses immediately with clean water (after plumage and evisceration) and change as frequently as possible (ideally after each batch of carcasses)  5.2 Wash knives and other cutting materials as often as possible  5.3 Store and transport carcasses in a covered container containing ice / freezer  5.4 Wash hands before handling carcasses upon arrival at the site and use clean containers for packaging  5.5 Do not keep raw carcasses and grilled chickens in the same freezer or with other types of products  5.6 Do not overload the freezer with carcasses and clean it regularly (twice a year) |
| Module 6. Preparation and Cutting | 6.1 Clean and disinfect the cutting table before and after cutting  6.2 Clean and disinfect the utensils used (knives, trays, forks) before and after cutting  6.3 Do not handle grilled chicken with your bare hands (use a fork and knife)  6.3 Avoid talking or sneezing during cutting if not wearing a mask or sneezing into the elbow crease when turning around |
| Module 7. Serving and seasoning | 7.1 Use non-perforated, intact and firm vegetables (especially tomatoes)  7.2 Wash, disinfect and keep vegetables free from contamination (flies, cockroaches, mice, rats and dust)  7.3 Properly store seasoning products (chili, salt, mustard, etc.)  7.4 Use clean and disinfected cutlery (trays, plates, spoon and fork) for service  7.5 Provide customers with clean water and liquid soap for hand washing |
| Module 8. Personal hygiene | 8.1 Employee health cards must be up to date  8.2 Have a handwashing device and wash your hands regularly with clean water and soap before and after each step of the chicken preparation process  8.3 Use clean work clothes (aprons, hats, towels and masks)  8.4 Do not allow any employee suffering from illness (diarrhea, vomiting, skin infection, cough, cold injury) to work  8.5 Ensure that all employees maintain hygiene (wash daily before starting and regularly cut nails) |
| Module 9. Environmental health and sanitation | 9.1 Choose a safe location for the outlet, safe from potential contaminants  9.2 Furnish the workplace with easy-to-maintain materials and colours  9.3 Keep the work environment clean (sweep, clean and disinfect)  9.4 Have a good drinking water supply and storage system  9.5 Have an adequate raised plunge and renew at least three times a day  9.6 Have an adequate garbage can (large, covered and washable), empty daily  9.7 Have a functional sewage disposal system  9.8 Maintain toilets (ensure cleanliness and disinfection of toilets daily)  9.9 Put a handwashing device at the entrance to the toilet |
