# Supplementary material for: Fast food over safe food? A qualitative evaluation of a food safety training intervention for street vendors applying the COM-B model in Ouagadougou, Burkina Faso
Source: PLoS One. 2024 Nov 21;19(11):e0313635. doi: 10.1371/journal.pone.0313635 (PMC11581311; doi:10.1371/journal.pone.0313635)
Supplement: S2 Table — (DOCX) [file pone.0313635.s002.docx]

**S2 Table. Interview guideline (with main questions)**

| Topic | Key questions |
| --- | --- |
| Introduction | Can you tell me a bit about your work and about your outlet? What is your role in the business, what are your responsibilities? |
| Food safety perceptions | What does food safety mean to you in the context of your work? |
|  | How do you feel about risks of food safety in the business you work in? |
|  | What type of food safety challenges do you experience during your work? |
| Training content & purpose | A few weeks ago, you attended a food safety training for vendors in Ouagadougou. When you think back to this time, what can you tell me about the training? |
|  | What did you think of the tools or equipment you received during the training for use at the outlet? |
|  | Could you give me some examples of things you learnt that were completely new to you? What about things that you already knew about? |
| Stories of Change | In your opinion: what positive or negative changes have you experienced after participating in the food safety training? A change can relate to anything! (e.g. Thoughts, beliefs, practices.) Everything you tell us is valuable. |
|  | What was [mention change] like before attending the training/ What motivated you to [mention change]? Or, what made it difficult to [mention change]? / What has supported this [change]? / How do you think this [mention change] compares to other vendors who did not receive this training? What do you believe is the impact of this [change] on you, your work, or on others? / How can you sustain this practice? Or what would make it difficult to sustain? |
|  | If the interviewee cannot think of ANY changes at all up to this moment: Could you tell me about the reason why you believe there have been no changes after attending the training? |
|  | Looking back at the last month, what do you think was – for you - the most significant change after participating in the training?  Can you share a short story/anecdote or example that captures this –most important change that has resulted from your or your employee’s participation in the training? Remember, this can be positive or negative, small or big, anything goes! Why is this story important to you? How do you think the training has contributed to this? |
|  | Can you think of any negative affects you have experienced because of this training? |
| Barriers & opportunities | Can you tell me a bit about the challenges you currently see when wanting to apply certain practices you learnt during the food safety training? Or that hinder safe food handling and preparation practices |
|  | What supporting resources are/can be provided to facilitate your application or use of safe food practices learnt during the food safety training? |
|  | Can you share your thoughts on the need/relevance of hygiene license and food safety health cards vendors are required to acquire? |
| Interaction | Can you tell me a bit more about chats you have with customers about food safety or hygiene? |
|  | When customers raise food safety concerns, how do you respond? How does that make you feel? |
| Closure | Any additions or questions |
