# Supplementary material for: Fast food over safe food? A qualitative evaluation of a food safety training intervention for street vendors applying the COM-B model in Ouagadougou, Burkina Faso
Source: PLoS One. 2024 Nov 21;19(11):e0313635. doi: 10.1371/journal.pone.0313635 (PMC11581311; doi:10.1371/journal.pone.0313635)
Supplement: S3 Table — (DOCX) [file pone.0313635.s003.docx]

**S3 Table. Coding scheme**

| Themes & Categories | Code | Example |
| --- | --- | --- |
| 1. Capabilities | | |
| *a. Psychological capabilities* | | |
|  | **Knowledge:** healthy chicken management | In terms of the cleanliness of the chickens that are alive, you have to clean the area well to avoid the chickens getting sick/ The cage … they had said that the sides should be able to let in air. |
|  | **Knowledge:** Handwashing | The training taught us to wash our hands before touching the chickens after handling the money. |
|  | **Knowledge:** Food Storage | The training brought us a lot of things. We learned that leftover grilled chicken should not be stored with fresh chicken and fish in the refrigerator. We didn't know this before. |
|  | **Knowledge:** Chicken preparation | Secondly, in terms of preparation also, how to maintain it and then protect the condiments as well. After grilling the chicken, how to protect it and go and give it to the customer too. |
|  | **Knowledge:** Clean cutlery use | Before [training] we held the chickens with our hand to cut them. Now we were advised to hold them with a fork and cut them. |
|  | **Knowledge:** Personal hygiene & health | If you yourself are clean, it motivates customers to comeback every day. |
|  | **Knowledge:** Healthy employees | Because during the training, we were made to understand that we, including our employees, should be in good health to present ourselves at the point of sale |
|  | **Knowledge:** Clean, washed vegetables | The training taught us a lot of things, so the proper use of onions, tomatoes, peppers... We apply all of this. |
|  | **Knowledge:** Hygienic working environment | You have to do the dishes well, clean where the chickens are prepared, also clean the glass container well to prevent flies from getting in, that's what hygiene concerns. |
|  | **Knowledge:** Hygienic slaughtering practices | At the training, we were told to pluck the chicken in hot water, wash it to get rid of the dirt before grilling. |
|  | **Knowledge:** Sources of contamination | Well, since at the training it was explained to us that the person who takes the money must not cut up the chicken. There you go, you can't grill it and then take the money, because the money is too dirty. |
|  | **Knowledge:** Seasoning & condiments | We simply grilled. Apart from the salt that we added, we did not add anything. But since we came back we now add spices, garlic… |
|  | **Knowledge:** Separating sites for different activities | The place where the chickens are slaughtered must be kept separate, the place where they are plucked must be separate and the place where they are cut up must also be separate. |
|  | **Knowledge:** Business skills | The aspect that I appreciated most during the training was financial management. Once you have income from your job, how to manage that income. Before, I was mixing everything up. Any profit I earned went straight back into the working capital to increase the capital. But during the training we were shown how to manage our income wisely. We were even made to understand that we had to estimate our salary on the job. These are things that we didn't do and didn't even think about. |
|  | **Awareness** of unsafe practices / wake-up call | Even if they had not given me tools, the training itself helped me because there are practices that I thought were good but in view of what we were shown at the training, they were rather inappropriate/wrong. In any case I can say that the training was very beneficial for me. |
|  | **Awareness** of unsafe practices / importance role of vendor | It's the dirt that can lead to diseases, so the risks will not be limited to the worker only but all the customers, so it's an obligation for the vendor to secure the space and the food in cleanliness so that if someone comes and sees it he is happy. |
|  | **Self-efficacy** (confidence in one’s food safety capabilities) | It's the ownership side that has become more pronounced at my level. From the slaughter until the chicken is consumable. Whoever eats it appreciates a job well done. |
|  | **Educational attainment** | During the training, we watched a video projection and afterwards we received a sheet on which we had to check off good hygiene practices and at the end, we received a training certificate. I didn't go to school, I don't know anything about paper business, so I can't venture into this field at the risk of making a mistake. |
|  | **Work experience** | When I say that we haven't studied, for example if you take this paper and ask me what it says, I don't know. But the experience I have in this work allows me to say that it [education] is not everything, because it is this [workplace] that I built and I got married, so if I say that it is not important, I lied. |
| *b. Physical capabilities* | | |
|  | Tool applicability (skills) | The cutting board that we got there; you see before I used to clean part of the table for cutting chicken. Now it's cutting on the board, and we clean up afterwards. |
|  | Usefulness of tools | The tools I was given, I have not done a single day without using them. |
|  | Ease of use of tools | With the new things, the work itself is easy, it has become easy. |
| 2. Motivation | | |
| *a. Reflective Motivation* | | |
|  | **Perceived** **responsibility** consumer & vendor health | What I remember is that they said that the vendors are the ones who feed the population the most, given the importance of their activity. |
|  | **Reputation**: retaining and attracting consumers | If a customer has a health problem after eating food bought here, he will not come back and he will go where it is clean, so if there is a risk, it will spoil our market and it will not be beneficial for us. |
|  | **Client satisfaction** | Customers' concerns are considered, we like it when they tell us to do this or not to do that. We also do what they want and they are happy. We listen to the clients. |
|  | **Higher profits** | Customers call me, if I go and listen to them, they say, ‘Hey, you have to send two chickens. It's because they think you're clean that they order up to two chickens. |
|  | **Regulatory Compliance** | Ha, as they don't even warn us before they [safety experts] arrive for their inspection, I would like them to be happy, even on the day they surprise me. |
|  | **Perceived consumer demand for safe food** | Yes, there are customers who control everything, from the choice of live chicken onwards. They judge the condition of the live chicken, the way it's going to be slaughtered, plucked, washed before they go in. In the whole process, if there is a treatment that doesn't suit him, he will let you know. If the water is dirty, some may give up and leave. |
|  | **Time** pressure (fast food over safe food) | It's the behaviour of some people who are in a hurry. For example, when we tell them not to touch the food directly or to put it on the cutting board, they say to do it quickly, they will leave. |
|  | **Consumer** **feedback** (Compliments and critic) | We accept (critic) it and reassure them that we are making efforts to change things. Someone who blames you for improving, you can only accept that” |
|  | **Intentions** | About the layout of the place, it was in our plans but with the motivation of the training it has only increased |
| *b. Automatic Motivation* | | |
|  | **Passion for work** | Immediately after training. When you work and you love your work when you are given advice to improve your work, you adopt it.” / “If I abandon the hygiene practices, it means that I don't want to work anymore. A *Mossi* adage says that: "if you don't want to stop farming, buy a new *daba*. |
|  | **Reinforcement** (exposure to reminders) | Yes, I want to redouble my efforts in this area. I want to do better than this. When I look at the certificate of the training I am really happy! |
|  | **Habits** | Washing my chickens, preserving my chickens, that's what I was always doing. In Papa's time, he told us if you slaughter the chickens, you finish and you wash them, with every ten chickens, you throw the water there; for every ten washed chickens you throw away the water. […] I used to do that already when I was a kid. |
|  | **Fear** | No, no, they said that if you want to wash the chicken, the third water bucket there, you have to use bleach. But we didn't take that [advice]. […] The dosage itself, I didn't follow. |
|  | **Faith** | We ask God to give us all longevity and that he blesses everything we do because it is the blessing that protects the whole earth. You can't achieve anything without the blessing. |
| 3. Opportunities (and barriers) | | |
| *a. Physical* | **Financial** resources | I would say the question of equipment. What the training requires us to put into practice in the development of our working environment is difficult because of the lack of our financial resources. |
|  | **High cost of living/uncertainty/economy** | You used to be able to get 1000 francs profit on a chicken. But with chickens these days (increased prices), some people are struggling to make 250 francs profit. |
|  | **Certificate/proof of good safety measures** | If you look at the windows, I had glued this [certificate] there and even the two sides are glued at my place and at the other small place. So when the customers arrive, they start reading straight away and they appreciate it a lot, so this makes you already happy. |
|  | **Market** **infrastructure & lay-out (dust, gutter, crowdedness)** | In relation to the gutters, because we had a lot of discussions on that because many people had said that it is not normal that we place ourselves on the gutters but in Ouagadougou if we take about one hundred vendors, those who are not on the gutters, it does not exceed five people. So […] we don't have the choice at first. So, if you look at where people are concentrated, the slabs are many and closed, so it's what can happen afterwards that we don't know. |
|  | **Regulatory environment** | If, for example, when they go to the sales site, if the foods are not well laid out, they can call us to order on the hygiene to be respected. Why don't they try to train us themselves? They are more interested in paying the money. For me, this is not a good way to change things. They should try to train us, that's what can change things. |
|  | **Continued training/learning** | I would urge them [trainers] not to give up on this work and to keep us in our sensitizations. If they see that we are starting to go off the rails, let them help us because a human being never finishes learning. |
| *b. Social* |  |  |
|  | **Social support** | Well, it wasn't me, it was someone who did the spending for me (outside help with the refurbishment of the outlet). I spoke to him, and he financed the work. For the fitting out, I know it was a bit expensive, but it's a gift. |
|  | **Competition vendors** | There are several things, winning the sales points is difficult as well as the means currently available to set up well. |
|  | **Outlet culture** | Because of that [employees’ poor attitudes toward safety] I am fighting with my employees. I had two employees and I recruited two more so that cleanliness is well maintained in my space. […] [Employees] have to be supervised otherwise at the slightest inattention they start to manipulate their mobile phones. In my presence they act *sap sap* (quickly) according to my instructions […] It's like that, if I'm not there, it's like usual.” |
|  | **Spill-over and teaching others** | The training really helped us in a sense that when you visit a colleague you can talk to him about the health risks related to his practices. So this salesman also benefits from the knowledge that he learned during the training. The training really helped in the work. |
|  | **Need for approval owner** | But it is difficult as an employee to say exactly what accommodation needs to be made because the decision and the means to do it rest with the boss. |
|  | **Fast employee turnover** vis a vis investments | The individual health record is a problem because you can recruit an employee and then you can get rid of them in a short period of time. In this situation when you recruit another one and the controllers come, they will ask the new employee's card. […] That's what really bothers us, in one hour you can hire and fire. We can pay but the employees don't stay. You can make the book by doing business and. a bit later you are obliged to thank the employee, in this case, it is a loss for you. |
| 4) Behavior changes | | |
|  | Cage maintenance | We have changed everything. Even the chicken coop is fixed every morning. It's clean. If it gets dirty, I have it washed. If there is chicken waste, I have it washed. |
|  | Buying new tools | Well, before we had only one table where we did everything, the plucking, the cutting, everything. As we were told during the training that this is not good hygiene practice, so my boss bought another one, a second table. |
|  | Training staff | We often have the chance to go to places that children don't have access to, so we must teach them. You do such and such, it's not normal in this job. But after the training, I showed them how they should do it too |
|  | Using fresh products | The chickens there, we leave them alive […] it's when the customer comes, that he chooses the chicken he wants and then we kill it. |
|  | Protecting outlet from sources of contamination | To prevent dust from getting in. You see the poles I made next to it, even if it rains, the water can't get in. |
|  | Personal hygiene | We wear masks (nose plugs) and we cover our heads with a cap when we work. |
|  | Change in food preparation style | For example, in the plucking and washing of the chickens, we used to do everything dry and then we used the flame to finish the plucking, then we opened it and grilled it. |
|  | Clean environment and sanitation | Well, in any case, with the woman [newly employed cleaner] now, cleaning is done each morning and evening. Before, it was only in the morning that we cleaned. Before that too, we had placed the rubbish bin close by. But now we put it behind (further away) |
|  | Handwashing | Well, a lot of things have changed. For example, we used to just give the customers plain water to wash their hands before eating. Now we never forget to give them soap. Others tell me that in my place it's good. Others tell me that at home it's fine, but elsewhere they often don't give soap to wash their hands unless they ask for it. |
|  | Renovation of facility | After the training, we did everything we could to get a window. Also, we only had two dishes, so we added two more to complete the four and we also use bleach […] we used to but not like that, but now we use bleach more than before.[…]We fixed that [glass]. |
|  | **Recruiting** new staff | Well, in terms of cleanliness, before it was us who cleaned, but as we went to the training, we hired a lady who now cleans, and even the oven, she cleans it too. |
|  | **Change in slaughtering practices** | Well! If I must prepare chicken, I wash it in four clean waters before I start preparing it. |
|  | Task division | I'm the cashier now, so I don't touch the meat. If I must touch it, it's with a fork |
|  | Clean cutlery use | Well, before, we used to use only our hands, we just rolled up the meat with the bag and cut it up; but now, as we have been made to understand that this is not a good practice, we have left it. |
