# Supplementary material for: Fast food over safe food? A qualitative evaluation of a food safety training intervention for street vendors applying the COM-B model in Ouagadougou, Burkina Faso
Source: PLoS One. 2024 Nov 21;19(11):e0313635. doi: 10.1371/journal.pone.0313635 (PMC11581311; doi:10.1371/journal.pone.0313635)
Supplement: Attachment — Submitted filename: PONE-D-24-30028_R1.Response to Reviewers.docx [file pone.0313635.s005.docx]

**PONE-D-24-30028 | Revision 1 | Rebuttal Letter**

**Fast food over safe food? A qualitative evaluation of a food safety training intervention for street vendors applying the COM-B model in Ouagadougou, Burkina Faso**

**Point-to-point response to the editor’s and reviewers’ comments (in *italic*)**

***

**Reviewer #1**:

***Comment 0****: The authors have conducted commendable work addressing the issue of food safety in chicken sales outlets in Ouagadougou, Burkina Faso. Through a qualitative study, they demonstrate the positive impact of training provided to vendors on improving their food safety practices. The results clearly indicate that the training was beneficial, leading to a significant improvement in vendors' hygiene practices. However, since this is an interventional study, it would have been helpful to include, in the introduction section, results from an assessment of the vendors' knowledge, attitudes, and practices prior to the training. This would offer readers a clearer understanding of the intervention's (training) impact on the improvement of food safety practices among vendors. I recommend publication after addressing the following minor corrections:*

**Response:** We greatly appreciate the reviewer's time and kind compliments on our manuscript. We agree that an overview of the assessment of vendor’s knowledge, attitudes and practices prior to the training strengthened the understanding of the training’s impact on food safety practices. Please see the phrases added to the introduction of the manuscript, on lines: 104-122. We have addressed your valuable corrections in detail below and hope this addressed your comment.

***Comment 1.*** *Harmonize the referencing system in the text [line 70 (1), (10) and line 78 (14,15)].*

**Response**: Thank you for capturing these errors. We have now carefully checked our references and ensured they are harmonized.

***Comment 2.*** *Remove the description of the COM-B model from the introduction and move it to the methodology section.*
**Response**: We agree with the reviewer that the description of the COM-B model better fits in the methodology section. We have therefore moved down this paragraph to the beginning of the Methods section, see lines 129-144.

***Comment 3.*** *Add a column to Table 1 to include the order number.*

**Response**: Thank you for this suggestion. We have now added a new column to table 1 which includes the order numbers for clarity.

***Comment 4.*** *Lines 228 to 233: I suggest replacing this verbatim with a testimonial illustrating the impact of psychological skills on improving food safety.*

**Response**: Thank you for the suggestion to replace the verbatim on the impact on financial / business skills with a testimonial illustrating the impact of psychological capabilities on food safety improvements. We have replaced this quote with the following testimonial: *Food safety, in my opinion, is mainly a matter of taking ownership, making sure that you don't get sick after preparing and eating the chicken” (Issa, employee)* Please see lines 265-267.

***Comment 5.*** *Line 267: Also provide prices in US dollars, and similarly for lines 271 and 272.*

**Response**: Thank you for this important comment. We have converted FCFA into US dollars on lines 314, 319 and 320, using the December 2022 exchange rate (added in between brackets on page 12).

**Reviewer 2**

***Comment 1.*** *The paper entitles “Fast food over safe food? A qualitative evaluation of a food safety training intervention for street vendors applying the COM-B model in Ouagadougou, Burkina Faso “is a really pleasant document to read. It deals with a qualitative analysis of the impact of training on the behavior of chicken sellers as well as owners of outlets, for a better awareness of the harmful effects that a lack of hygiene could cause. This is a very important topic cause; lack of food safety can negatively impact public health specially concerning ready-to-eat food. More importantly, after the training, evidence can be observed in the field and the testimonies of vendors and owners justify the need to repeat such kind of training. The scientific way (ethical authorization and vendors and owner consent) the topic is treated involve COM-B methods and is conducted with a good manner and with evidences. Therefore, I recommend it publication with one small recommendation that does not affect the paper quality. It should be interesting to emphasis that selling ready-to-eat chicken is and activity devoted to men.*

**Response.** We are grateful for the positive feedback of reviewer 2 on our manuscript. Thank you very much for highlighting the important observation that selling ready-to-eat chicken is mainly an activity in which men are involved. We have now emphasized that this is predominantly a men’s activity in this context, see line 150, and backed this up with the following reference:

Gemeda BA, Dione M, Ilboudo G, Assefa A, Lallogo V, Grace D, Knight-Jones TJ. Food safety and hygiene knowledge, attitudes and practices in street restaurants selling chicken in Ouagadougou, Burkina Faso. Frontiers in Sustainable Food Systems. 2024;8:1448127.
